# Integrating Micro- and Nanostructured Platforms and Biological Drugs to Enhance Biomaterial-Based Bone Regeneration Strategies

**DOI:** 10.1021/acs.biomac.4c01133

**Published:** 2024-12-02

**Authors:** Syed Ahmed Shah, Muhammad Sohail, Paweł Nakielski, Chiara Rinoldi, Seyed Shahrooz Zargarian, Alicja Kosik-Kozioł, Yasamin Ziai, Mohammad Ali Haghighat Bayan, Anna Zakrzewska, Daniel Rybak, Magdalena Bartolewska, Filippo Pierini

**Affiliations:** † Department of Biosystems and Soft Matter, Institute of Fundamental Technological Research, Polish Academy of Sciences, Warsaw 02-106, Poland; ‡ Faculty of Pharmacy, The Superior University, Lahore 54000, Punjab, Pakistan; § Faculty of Pharmacy, Cyprus International University, Nicosia 99258, North Cyprus

## Abstract

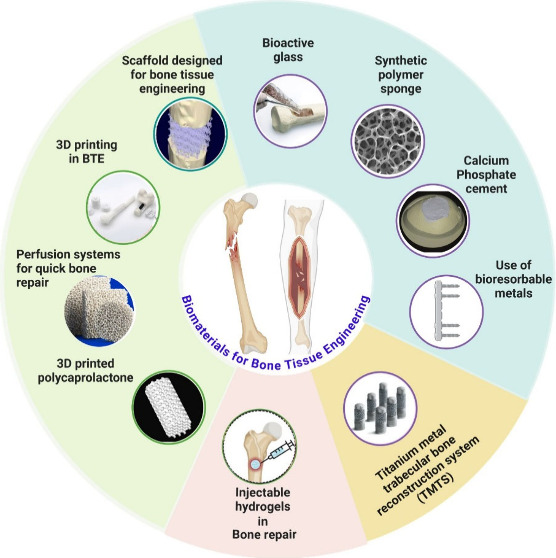

Bone defects resulting from congenital anomalies and trauma pose significant clinical challenges for orthopedics surgeries, where bone tissue engineering (BTE) aims to address these challenges by repairing defects that fail to heal spontaneously. Despite numerous advances, BTE still faces several challenges, i.e., difficulties in detecting and tracking implanted cells, high costs, and regulatory approval hurdles. Biomaterials promise to revolutionize bone grafting procedures, heralding a new era of regenerative medicine and advancing patient outcomes worldwide. Specifically, novel bioactive biomaterials have been developed that promote cell adhesion, proliferation, and differentiation and have osteoconductive and osteoinductive characteristics, stimulating tissue regeneration and repair, particularly in complex skeletal defects caused by trauma, degeneration, and neoplasia. A wide array of biological therapeutics for bone regeneration have emerged, drawing from the diverse spectrum of gene therapy, immune cell interactions, and RNA molecules. This review will provide insights into the current state and potential of future strategies for bone regeneration.

## 1 Introduction

**Figure 1:**
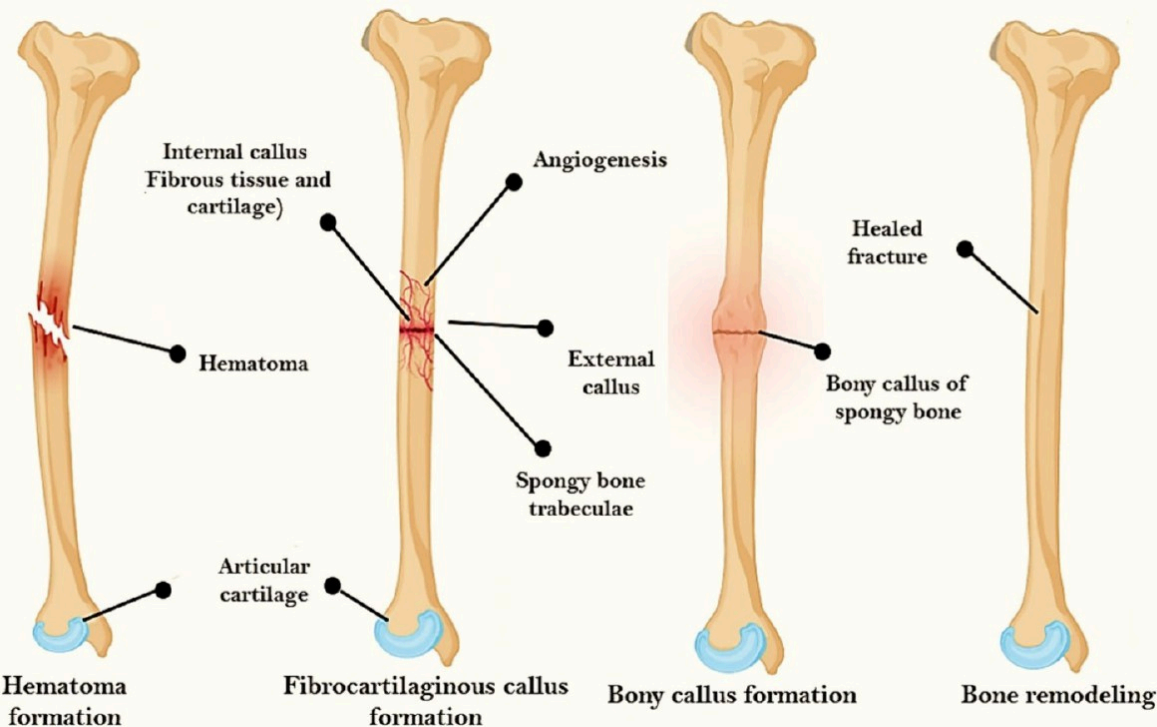
Illustration depicting the sequential stages of the bone healing process. From left to right: Hematoma stage characterized by blood clot formation at the fracture site; Fibrocartilaginous callus formation indicating the initial bridging of the fracture gap with fibrous tissue; Bony callus formation depicting the progression toward the development of mature bone tissue; Remodeling stage showing the gradual restoration of bone structure and alignment.

Bone tissue possesses remarkable regenerative capabilities, healing most fractures without intervention. This regeneration process involves a coordinated series of biological events orchestrated by various cell types and molecular signaling pathways.^1^ In clinical settings, the most common scenario for bone regeneration is fracture healing, which mirrors the developmental processes seen in fatal skeletal growth. Bone injuries typically heal without scar tissue formation, unlike other tissues.^2^ Instead, the regenerated bone closely resembles its original state, with properties primarily restored. However, there are instances where bone regeneration is impaired, particularly in challenging cases like tibial fractures and older or obese people.^3^ Furthermore, there are orthopedic and oral/maxillofacial surgery situations where substantial bone regeneration is necessary, surpassing the body’s natural healing capacity. This includes cases of significant bone defects resulting from trauma, infection, tumor removal, or skeletal abnormalities, as well as conditions like avascular necrosis and osteoporosis, where the regenerative process is compromised.^4^ The process of bone fracture repair reflects embryonic bone formation, progressing through four distinct phases, as shown in Figure 1. The initial phase, inflammation, rapidly forms a blood clot at the fracture site, attracting phagocytic cells through chemotaxis.^5^ This stage relies on adaptive and innate immune responses, with mesenchymal stem cells (MSCs) playing a crucial role in maintaining immune balance by releasing immunosuppressive factors.^6^ Subsequently, the repair phase commences as osteoblasts cover the clot, proliferating intensely while MSCs migrate and differentiate into osteoblasts.^6,7^ In cases of mechanical instability, MSCs differentiate into chondrocytes, forming a bone callus to stabilize the fracture site, marked by extracellular matrix mineralization. Finally, in the remodeling phase, catabolic activity reduces callus volume through cartilage resorption while angiogenesis continues, ultimately resulting in lamellar bone formation.^8^ Alongside stem cells, various growth factors and signaling agents actively participate in bone tissue restoration. Successful bone tissue repair entails the growth of damaged extremities without scar formation, emphasizing the importance of the healing process.

Bone mechanotransduction plays a vital role in tissue regeneration, with traction contributing to osteogenesis and the distension of surrounding tissues.^12^ Mechanical modulation through hydrostatic pressure and traction tension influences the repair process, stimulating regeneration. In cases requiring intervention, osseointegration validates surgical implants, facilitating a connection between the implant and living bone tissue. Implants gradually replace injured tissue, providing functional support for load-bearing.^9^ Fracture fixation and immobilization influence the differentiation of osteogenic stem cells, determining whether chondrocytes or osteoblasts form. Given these complexities, developing products that enhance stem cell survival, signaling factors, and osteoinductive substances is crucial.^10^ This understanding paves the way for new discoveries in bone tissue engineering, offering promising avenues for bone repair and localized and systemic therapies. To address such complexities, a diverse arsenal of bone graft materials is available, including autologous bone (harvested from the same patient), allogeneic bone (sourced from donors), demineralized bone matrices, and a broad spectrum of synthetic bone substitute biomaterials, ranging from metals and ceramics to polymers and composite materials.^11^ In addition to conventional methods, biomaterials for tissue regeneration and repair have emerged as a beacon of hope. Researchers are delving into innovative materials and techniques that mimic natural bone properties, opening new avenues for enhancing bone regeneration and surmounting the limitations of current grafting methods.^12^ These biomaterials hold the promise of revolutionizing bone grafting procedures, indicating a new era of regenerative medicine and advancing outcomes for patients worldwide.^13^ Scaffolds and hydrogels, commonly used biomaterials, provide a temporary matrix to support cell migration and capillary growth. The architecture of these scaffolds is crucial, as it can significantly influence vascularization effectiveness.^14^ The chemical composition of these biomaterials is another critical factor. It directly affects endothelial cell interactions during vessel formation, with certain materials showing proangiogenic properties that facilitate neovascularization and bone regeneration. Biomaterials like fibrin, heparan sulfate, and hydroxyapatite play a role in vascularization by binding to angiogenic cytokines and enhancing growth factor activity at defect sites.^15^ Additionally, recent developments have focused on biomaterials capable of interacting with growth factors. These include synthetic biomaterials modified with heparin-binding peptides and other substances that can sequester and amplify growth factor activity, thereby improving vascularization and bone regeneration.^16,17^

## 2 Bone Tissue Engineering through Time: A Historical Overview

The quest to find optimal solutions for replacing lost bone and developing superior bone replacement materials has been a timeless pursuit of humanity. Archaeological marvels, like the adorned Incan skulls with gold^18^ and silver plates concealing defects provide intriguing insights into ancient civilizations’ early endeavors in bone repair.^19^ Similarly, ancient Egyptians showcased advanced orthopedic and traumatological procedures, with surgeons performing knee joint replacements as early as 600 BC using iron prostheses. In the modern era, Dutch surgeon Job Janszoon van Meekeren etched his name in history with the pioneering bone xenograft procedure in 1668.^20,21^ This groundbreaking method successfully treated a skull defect in a Russian nobleman by utilizing a bone xenograft extracted from a deceased dog’s calvaria, seamlessly integrating into the patient’s skull. In subsequent centuries, they witnessed the emergence of various techniques, ranging from plaster of Paris to ivory cylinders, aimed at addressing bone cavities and defects.^22^ Pioneers like Louis Léopold Ollier and Arthur Barth significantly contributed to the evolution of modern bone grafting procedures in the late 1800s. Ollier’s groundbreaking experiments on bone formation in animal models and Barth’s meticulous histological assessments laid the essential groundwork for contemporary bone grafting techniques.^23,24^

**Figure 2:**
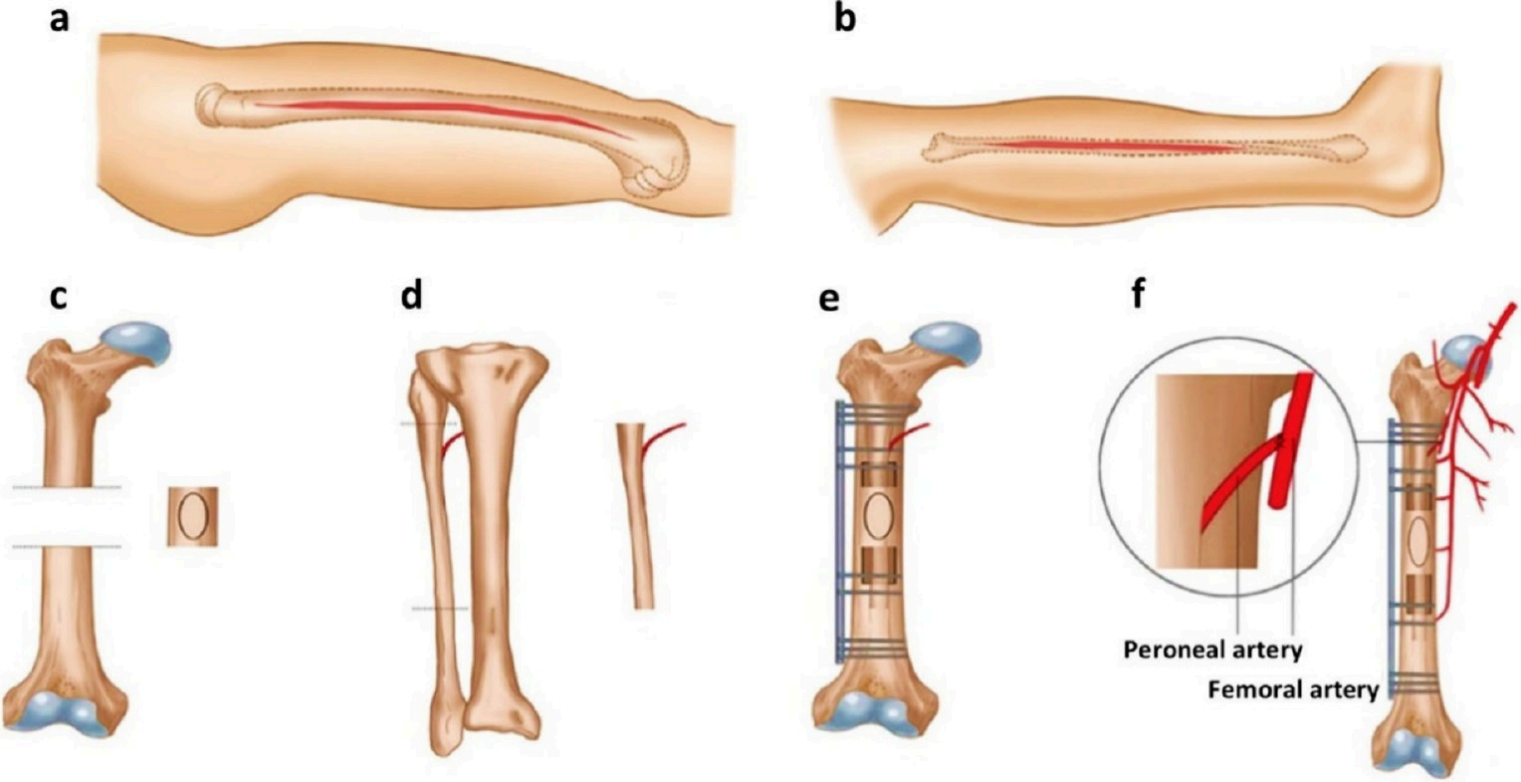
Pictorial representation of allograft and autograft in intramedullary femoral defect and representing the multiple surgeries in bone defects model. (a) The lateral femur approach for the excision of the tumor. (b) Utilizing the lateral fibula approach, a vascularized fibula was obtained. (c) A wide excision of the tumor was conducted, followed by pasteurization of the specimen in saline preheated at 65 °C for 45 min. (d) Harvesting of the vascularized fibula was performed. (e) The pasteurized bone with the vascularized fibula in its medullary canal was positioned into the original anatomical site and secured with a plate. (f) Microscopic anastomosis was carried out and recreated from ref (25).

In the 20th century, they witnessed a surge in bone graft demand, driven by advancements in orthopedic techniques and joint replacement procedures. The establishment of the first bone bank for allogenic bone grafts in New York in 1945^26^ marked a monumental milestone, albeit accompanied by concerns regarding immunological reactions to transplanted allogenic bone material, illustrated in Figure 2. Despite strides in bone substitute materials, autologous bone grafting remains the gold standard due to its unparalleled properties for bone regeneration.^27^ However, autologous bone grafts pose limitations, including donor site morbidity and limited volume availability, particularly for treating significant bone defects.^28,29^ Allografts, while addressing some of the autologous grafts’ limitations, present their challenges. The risk of immunological reactions and the processing steps required to remove antigenicity often render allografts inferior to autologous graft options.^28^ Alternative approaches like the Masquelet technique offer promising solutions by harnessing the body’s immune response to promote bone reconstruction. However, they still rely on autologous bone grafts, underscoring the ongoing need for innovative solutions in bone grafting.^30,31^ Looking ahead, technological advancements and surgical procedures continue to broaden the horizons for bone grafting materials. With the global population aging and the demand for joint replacements rising, the bone grafting market is poised for steady growth, propelling further innovation in the field.^32^

**Figure 3:**
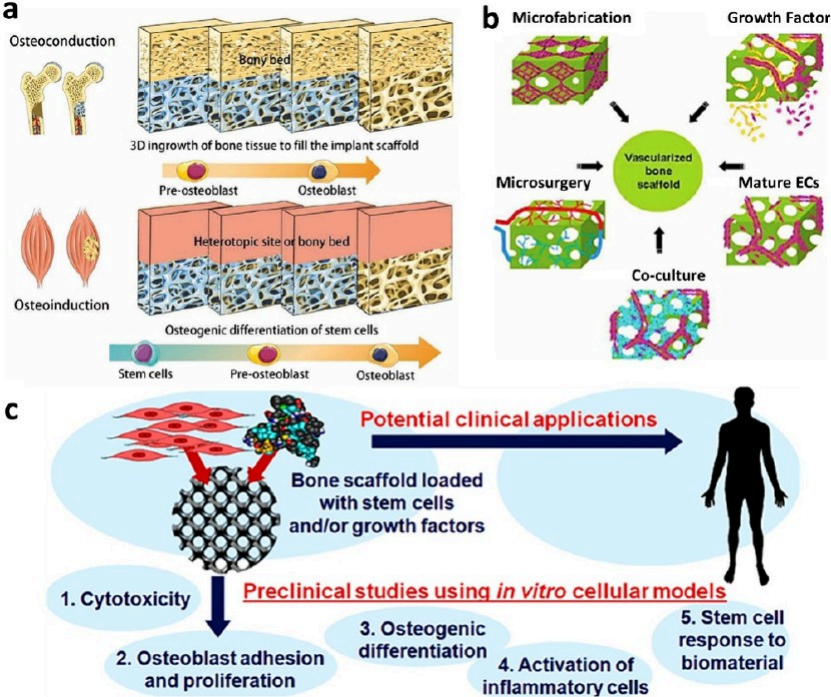
Role of biomaterials in bone tissue regeneration. (a) concepts of osteoconduction and osteoinduction.^50^ (b) Vascularization by bone scaffolds, showing the growth factors involved in revascularization and synergistically promoting bone regeneration.^52^ (c) Cell-biomaterial interactions loaded with stem cells to assess potential clinical applications and evaluate cytotoxicity and other ex vivo preclinical studies.^54^

Progress in materials science and nanotechnology has provided tissue engineers with precious tools for directing cell behaviors in tissue formation. This advancement has been pivotal in enhancing the capabilities of tissue engineers to manipulate cellular environments at a microscopic level, significantly contributing to the development of more effective tissue engineering strategies.^33,34^ The role of biomaterials in different aspects of tissue regeneration has been investigated on various levels. Osteoconductivity, essential for bone regeneration, enables new bone formation on biomaterial surfaces. This vital aspect involves critical processes such as osteoprogenitor cell migration, proliferation, differentiation, and extracellular matrix deposition in bone defects.^35,36^ A crucial element of osteoconductivity is the formation of a carbonated hydroxyapatite layer on biomaterials, facilitating protein adsorption, cell attachment, and bone matrix deposition.^37^ This property significantly influences the integration of new bone with existing bone or implants, a fundamental factor in successful bone regeneration,^38^ as shown in Figure 3a. The osteoconductive properties of biomaterials are heavily dependent on their physicochemical characteristics, including chemical composition, surface properties, and geometry.^39^ Materials like calcium phosphate-based ceramics, bioglass, and Type I collagen are known for their excellent osteoconductivity due to their composition and structure.^40^ Additionally, nonbiological materials such as metals, ceramics, and synthetic polymers can be osteoconductive through coating or composite formation.^41^ For instance, titanium can be rendered osteoconductive with surface treatments,^42^ and synthetic polymers can gain osteoconductivity through composites with calcium phosphate ceramics.^43^ Another critical process in bone regeneration, osteoinduction, is where biomaterials stimulate primitive cells to develop into bone-forming cells.^44^ Osteoinductive materials impact ectopic bone formation at various levels, as shown in Figure 3a. At the tissue level, they facilitate vital functions like nutrition and oxygen exchange and promote vascularization necessary for tissue growth.^45^ Cellularly, they trigger stem cells to differentiate into an osteogenic lineage by forming a biological carbonated apatite layer.^46^ Molecularly, these materials concentrate on osteogenic proteins, enhancing local growth factor enrichment and stimulating cellular activities.^47^ Calcium phosphate-based bioceramics, such as hydroxyapatite and tricalcium phosphate, are widely used for their osteoinductive properties attributed to their calcium and phosphate content.^48^ However, other materials like poly(hydroxyethyl methacrylate), alumina ceramic, and titanium, although lacking calcium phosphate, have also exhibited osteoinductive properties under certain conditions, highlighting the importance of chemical composition. A critical feature of osteoinductive materials is their porous macrostructure. Bone induction mainly occurs in the pores of implants, where ions precipitate after reaching supersaturation. Microstructure, including roughness and porosity, also significantly influences osteoinductivity, as evidenced by different levels of bone induction in varied implant textures, given in Figure 3b. For instance, treated titanium implants with a microporous structure induce bone formation, unlike untreated titanium.^49^ The concepts of osteoconduction and osteoinduction are shown in Figure 3a.^50^ Another critical aspect, vascularization, is essential in bone regeneration, particularly for tissues exceeding 200 μm, the limit for oxygen diffusion *in vivo*. It involves the formation of blood vessels that integrate with the host’s blood supply, ensuring nutrient and oxygen delivery to cells and facilitating waste removal. This process also recruits progenitor cells for tissue regeneration (Figure 3b).^51,52^ However, natural vascularization in bone defects postinjury is often insufficiently rapid, leading to potential nutrient deficiencies and hypoxia, which can hinder bone healing. In response, biomaterials designed to promote vessel network formation have become integral in bone regenerative engineering.^53^

Biomaterials influence bone regeneration primarily through interactions between cells and biomaterial surfaces, with cell adhesion playing a central role.^55^ This adhesion, mediated by integrins (heterodimeric receptors on cell membranes), links cells to substrates by binding to adhesive proteins on biomaterial surfaces. Integrin-mediated cell adhesion is crucial for determining cell behaviors like morphology, mobility, proliferation, and differentiation.^54,56^ Integrins interact with the actin cytoskeleton upon binding to form focal adhesions, influencing cell morphology and fate. These cellular interactions largely occur at the biomaterial surface, where characteristics like chemical composition, hydrophilicity, and topography are key factors controlling cell behaviors.^57,58^ Biomaterials play a significant role in this process, serving not only as scaffolds for cell infiltration and tissue deposition but also providing inductive signals for tissue connection with host networks like the vasculature and nervous system.^59^ Initially, scaffolds support cell adhesion, and their porous structure facilitates nutrient and oxygen diffusion, enabling cell migration and proliferation within the scaffold. Biomaterials with suitable chemical composition and microstructure support vascular formation and stabilization. As the process progresses, osteoblasts deposit large amounts of tissue matrix, including collagen and minerals, along the scaffold structure. This newly formed ECM then undergoes a remodeling process, primarily mediated by osteoclasts, to integrate with the natural ECM. Scaffold degradation, timed with the remodeling process, is key to integrating new bone with the host bone tissue. Various strategies have been developed to enhance this integration in bone regenerative engineering.^60^ One such approach is to design scaffold porosity and architecture to improve nutrient and oxygen transport. Modifying the chemical composition of biomaterials through techniques like grafting, coating, and patterning, as well as the introduction of cell adhesive molecules, have shown promising results in improving tissue integration. Another innovative approach involves incorporating biological components into scaffolds to enable cell-mediated remodeling.^61^

### 2.1 First-Generation Biomaterials in Bone Repair

The first generation of biomaterials, primarily bioinert, includes metals like titanium and its alloys and porous tantalum, known for their strength, durability, and biocompatibility. These materials revolutionized bone repair and joint replacement surgeries.^62^ Poly methyl methacrylate bone cement, another first-generation material, became widely used for its immediate structural support and ease of application despite its nonbiodegradability and thermal necrosis risk. Titanium and its alloys stand out for their unparalleled biocompatibility and mechanical strengths.^63,64^ Due to these attributes, titanium is predominantly chosen for orthopedic implants among various metals. Titanium implants are typically anchored using cemented fixation, as seen in traditional hip replacements, or through direct bone ingrowth in cementless hip replacements.^65^ The latter method relies on a process known as osseointegration, where bone tissue forms directly on the titanium implant. The osseointegration is critical for the implant’s longevity, hinging on a dynamic bone-implant interface.^66^ When osseointegration is successful, this interface becomes densely populated with bone tissue, securing the implant firmly. A significant challenge faced by metal orthopedic devices, including titanium implants, is biocorrosion, which can produce considerable amounts of wear particles and metal ions.^67^ These ions stimulate the immune system and bone metabolism through various direct and indirect pathways, contributing to the pathophysiology of aseptic loosening. This issue is particularly significant considering that, despite the high success rates of cementless titanium implants, which stand at about 85% over ten years, this figure drops to 70% at the 15-year mark.^68^ Moreover, aseptic loosening leads to impaired implant fixation, resulting in pain and instability that are exacerbated by physical activity and weight-bearing.^69^ To address these concerns, newer titanium alloys such as Ti6Al-7Nb^70^ and Ti-13Nb13Zr,^71^ along with advanced fabrication techniques, including laser sintering, three-dimensional (3D) printing,^72^ and electrochemical anodization for creating nanoporous surface^73^ have been introduced, offering safer alternatives to the traditional alloys, specifically Ti-6Al-4 V,^74^ by eliminating potentially toxic vanadium and aluminum. These advancements, alongside preoperative Lymphocyte Transformation Tests for patients with known metal sensitivity, are crucial in enhancing titanium-based orthopedic implants’ safety and efficacy.^75^ On the other hand, a titanium metal trabecular bone reconstruction system (TMTS) has been used to reconstruct significant bone defects.^76,77^ TMTS is made of a porous titanium alloy with a microstructure miming the natural trabecular bone structure. This allows bone cells to easily attach to the scaffold and begin to grow into it. TMTS is a strong and durable material that can withstand the stresses of everyday life. It is also biocompatible, meaning it will not cause an immune response. In a recent study, researchers used electron beam melting technology (EBMT) to create a 3D-printed Tissue-Matched Temporary Biomaterial Resorption Systems (TMTBRS) implant, which was then evaluated in a clinical trial involving 30 patients with early osteonecrosis of the femoral head (ONFH).^76^ The TMTBRS implants were implanted into the patient’s femoral heads and followed up for 6, 12, and 24 months. A radiological examination was performed at each follow-up visit to assess the stability of the implants and the growth of bone into the trabecular holder portion of the implants. The study results showed that the TMTBRS implants were safe and effective.

**Figure 4:**
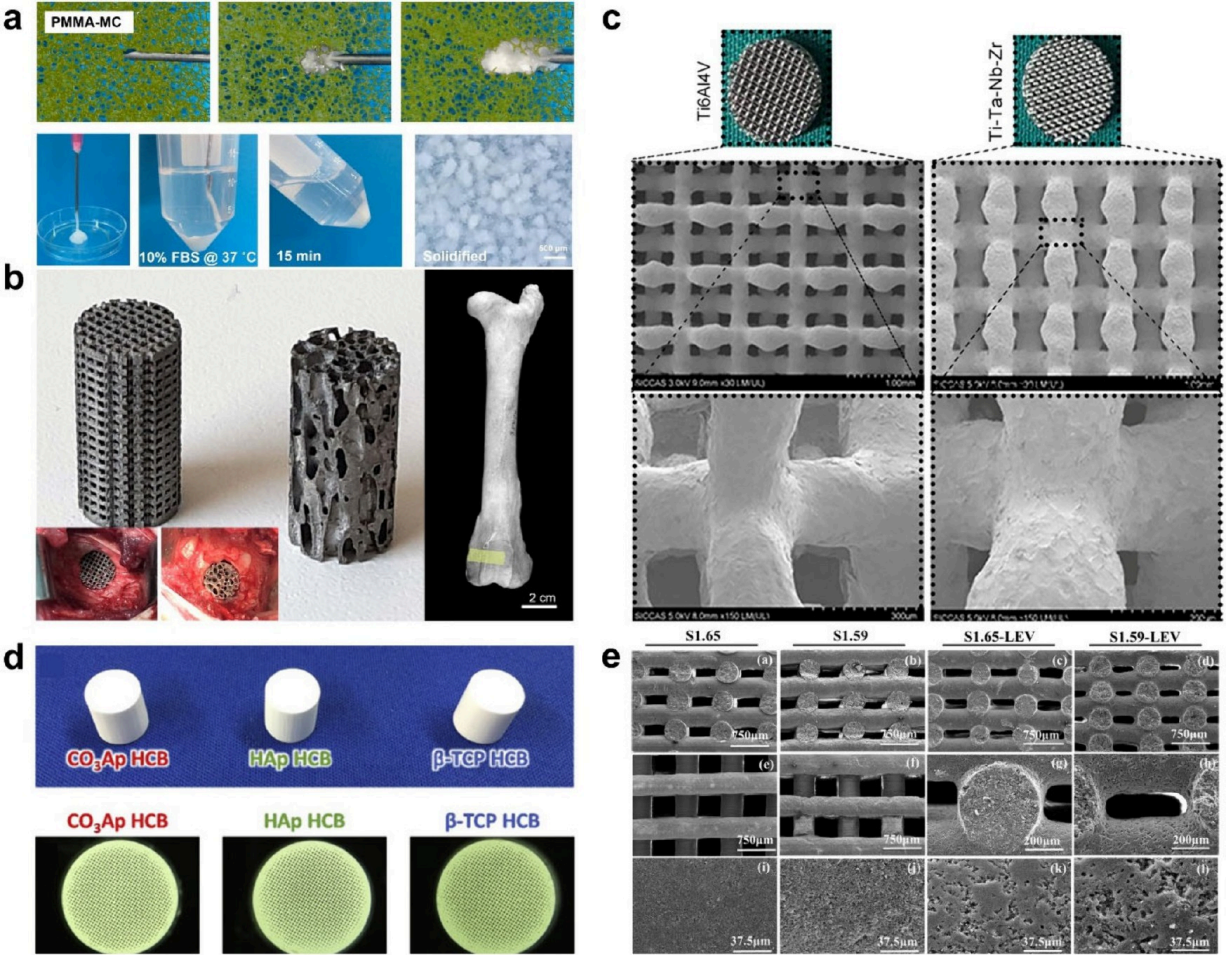
Showcasing biomaterials from both the first and second generations for bone regenerative engineering. (a) Overcoming the limitations of PMMA for bone regenerative engineering: The potential of PMMA/mineralized collagen (PMMA-MC) as a biomaterial for clinical hip replacement, particularly in osteoporotic conditions, by facilitating better osteointegration and mechanical stability. The injectability of PMMA-MC for prosthesis fixation is highlighted. The effective retention of PMMA-MC within a polystyrene sponge is shown, showcasing its improved injectability compared to conventional PMMA. The solidification process of PMMA-MC happened in Dulbecco’s modified Eagle’s medium (DMEM) supplemented with 10% (v/v) fetal bovine serum (FBS) at 37 °C.^88^ (b) Evaluating the impact of ADM technique (3D printing/rapid prototyping) on osseointegration in titanium implants. Titanium cylinders showcase two distinct microarchitectures: geometric (left) and trabecular (right). The surgical implantation site in the femoral condyle is indicated in yellow. The surgical placement of these implants in the ewe, with the geometric microarchitecture on the right and the trabecular microarchitecture on the left, is shown in the inset.^115^ (c) Fabrication of porous titanium–tantalum-niobium–zirconium scaffold (Ti–Ta–Nb–Zr) using SLM technology^94^ (d) The advantages of carbonate apatite (CO3Ap) over HAp and β-TCP, and their effects on bone formation and maturation. The photograph of the CO3Ap, HAp, and β-TCP with Honeycomb Blocks (HCBs) is displayed above, alongside stereomicroscope images of each type below^110^ (e) Fabrication of sintering-free biphasic calcium phosphate (BCP)/natural polymer composite scaffolds using robocasting, an additive manufacturing technique.^116^

Poly(methyl methacrylate) (PMMA) is another first-generation biomaterial for bone tissue engineering. This synthetic acrylic polymer has a proven track record in various biomedical fields and has gained significant traction in bone regenerative engineering.^78,79^ Its adoption in this domain is primarily due to its biocompatibility, mechanical robustness, and ease of fabrication, making it a common choice for bone graft substitutes, scaffolds, and fillers.^80,81^ However, PMMA’s application in bone regenerative engineering is a challenge. As a bioinert material, PMMA does not form chemical or biological bonds with the host bone at the implant surface, typically resulting in coverage by fibrous tissue without osteointegration at the implant site.^82^ This development of a fibrous tissue layer, similar to the problematic zone seen at the cement-bone interface in titanium implants, often leads to aseptic loosening. Additionally, the compressive elastic modulus of PMMA is significantly higher than that of the natural human vertebral body, posing another challenge.^83^ Researchers explored various strategies to enhance PMMA’s efficacy in bone regeneration to address these limitations. Surface modifications with bioactive molecules like hydroxyapatite,^84^ chitosan,^85^ or collagen^86,87^ have shown promise in improving osteoconductivity and promoting bone cell attachment and growth. For instance, in a recent study, researchers sought to enhance the bone-bonding ability of PMMA by incorporating mineralized collagen (MC) into the material (Figure 4A).^88^*In vitro* experiments demonstrated that PMMA-MC exhibited improved wettability and dynamic mechanical properties compared to pure PMMA. They also evaluated the impact of PMMA-MC on osteoporotic bone marrow stromal cells (BMMSCs). The results revealed that the addition of MC significantly promoted osteoblastic gene expression and suppressed adipogenic marker expression, which indicates the ability of PMMA-MC to stimulate bone cell differentiation and inhibit the formation of fatty tissue, which are crucial for bone regeneration. Moreover, combining PMMA with other materials, such as nanosilver and bioactive glass,^89^ has been investigated recently to develop more biocompatible and osteoconductive composites. These advancements aim to optimize PMMA’s functionality and integration in bone regenerative applications.

Among the first-generation biomaterials for bone tissue engineering, absorbable metal scaffolds (AMSs) represent a class of materials that offer the dual advantages of providing mechanical support during early bone regeneration and seamlessly integrating with the surrounding tissue as they biodegrade.^90^ Notably, magnesium (Mg) alloys have emerged as promising AMS materials due to their excellent biocompatibility, osteoconductivity, and structural similarity to natural bone.^90,91^ However, the uncontrolled degradation rate of Mg alloys has hindered their clinical translation. Researchers are actively exploring strategies to regulate the degradation rate to address this challenge, such as incorporating bioactive molecules or modifying the scaffold’s surface properties. Other advanced subcategories of ADM are Electron beam melting (EBM) and selective laser melting (SLM).^92,93^ Guo et al. investigated porous titanium–tantalum-niobium–zirconium scaffolds fabricated using SLM technology (Figure 4C).^94^ It has been mentioned that porous tantalum can promote bone regeneration. This material, another first-generation biomaterial, has been widely employed owing to its high porosity and excellent biocompatibility. The scaffolds developed in the Guo study had superior mechanical properties and enhanced osteogenesis compared to traditional scaffolds, demonstrating the potential of porous tantalum in repairing bone defects.

### 2.2 Second-Generation Biomaterials in Bone Regeneration

The second generation of biomaterials for bone regeneration has expanded the range of materials available for tissue engineering applications. Their bioresorbable and bioactive attributes have paved the way for developing more effective and durable bone tissue engineering strategies.^95,96^ Compared to their first-generation counterparts, these materials offer improved biocompatibility, osteoconductivity, and mechanical properties. Among them, biodegradable polymers play a crucial role in bone tissue engineering because they provide a scaffold for cell attachment, proliferation, and differentiation. Synthetic polymers, such as polylactic acid (PLA),^97^ polyglycolic acid (PGA),^98^ and polycaprolactone (PCL),^99^ offer the advantage of controllable degradation rates, which can be tailored to match the rate of bone regeneration. Naturally derived polymers, such as collagen and hyaluronic acid, provide innate biological guidance to cells, promoting bone formation.^100,101^ However, natural polymers often exhibit batch-to-batch variation and variable degradation rates. Bioactive glasses are a class of materials that form a direct chemical bond with bone, promoting osteoconductivity and enhancing bone regeneration.^102^ The first artificial bioactive material, 45S5 Bioglass, was developed in the late 60s by Larry Hench.^103^ This material has been used extensively in dental and orthopedic applications due to its excellent biocompatibility and ability to induce bone formation. Since the early 2000s, borate bioactive glasses (BBGs) have gained particular attention due to their superior bioactivity and bone healing capacity compared to silicate glasses.^104^ BBGs exhibit excellent biocompatibility, allowing them to interact favorably with living tissues, and they induce the formation of a calcium phosphate layer on their surface, which is similar to the mineral phase of bone. This bioactive layer promotes bone tissue formation and integration, enhancing bone regeneration. The next class of second-generation biomaterials for bone regeneration is calcium phosphate ceramics. These materials, such as hydroxyapatite (HAp), β-TCP, and Carbonate apatite (CO3Ap), are closely related to the mineral phase of bone, making them ideal candidates for bone tissue engineering. HAp is a highly biocompatible material that is well-accepted by the body and promotes bone formation, but it has a slow degradation rate.^105^ β-TCP is another naturally occurring calcium phosphate mineral with the chemical formula Ca3(PO4)2. It is a less biocompatible material than HAp, but it degrades more rapidly.^106^ β-TCP is often used in combination with HAp to achieve a balance of bioactivity and degradation rate.^107,108^ CO3Ap is another calcium phosphate mineral similar to HAp in composition, but it contains carbonate ions (CO32-) in its structure. CO3Ap is a biocompatible material that the body accepts and promotes bone formation. It is also resorbed faster than HAp, which may make it a more effective scaffold material for bone regeneration.^109^ A recent study has shown that CO3Ap can promote bone formation more effectively than HA or β-TCP.^110^ This study compared the effects of three types of honeycomb blocks (HCBs), composed of HA, β-TCP, and CO3Ap, on bone formation and maturation (Figure 4D). The HCBs had similar macroporous structures and compressive strengths, but the CO3Ap HCBs induced significantly faster bone maturation than the HAp or β-TCP HCBs. The authors attributed this difference to the disparity in calcium ion concentrations surrounding the HCBs. CO3Ap is resorbed only by osteoclastic resorption, while HAp is not resorbed, and β-TCP is rapidly dissolved without osteoclasts. This suggests that the controlled degradation of CO3Ap may provide a more favorable bone formation environment than HAp or β-TCP.

While polymers offer flexibility in degradation rates and bioactivity, ceramics possess excellent biocompatibility and structural similarity to bone minerals. However, polymers and ceramics alone can only partially optimize the requirements for bone regeneration. This is where nanocomposite biomaterials come into play. Nanocomposites combine the strengths of both polymers and ceramics by embedding nanosized ceramic particles within a polymer matrix.^111−113^ This hybrid material effectively bridges the gap between the flexibility and bioactivity of polymers and the excellent biocompatibility and mechanical properties of ceramics. The nanosized ceramic particles provide a higher surface area for cell attachment and proliferation, while the polymer matrix facilitates degradation and integration with the surrounding tissue. For instance, a recent study has demonstrated the potential of strontium-containing HAp (SrHAp) nanoparticles embedded in PCL scaffolds for bone regeneration. This study showed that the PCL/SrHAp composite scaffold promoted cell proliferation and osteogenic differentiation of rat bone marrow-derived mesenchymal stem cells (BMSCs). *In vivo* experiments further revealed that the PCL/SrHAp scaffold could stimulate bone regeneration in a cranial defect model. This study highlights the potential of nanocomposites for bone tissue engineering applications. The selection of nanocomposites is crucial for specific bone defects, such as craniofacial defects.^114^ Incorporating growth factors into nanocomposites can further enhance bone regeneration by regulating the release of these factors and controlling new bone generation. Ongoing research is exploring novel nanocomposite formulations, fabrication techniques, and applications to advance their therapeutic potential in regenerative medicine further.

## 3 Medical Implants and Scaffolds for Synergistic Bone Healing

One of these strategies is additive manufacturing (ADM), a rapidly evolving technology that has revolutionized the fabrication of medical implants and scaffolds.^117,118^ ADM allows for the creation of complex and customized structures that closely mimic the natural architecture of bone, providing optimal conditions for cell adhesion, proliferation, and differentiation.^119^ In a recent study, researchers demonstrated the use of robotically aided printing (robocasting) to fabricate sintering-free BCP/natural polymer composite scaffolds for bone regeneration (Figure 4E).^116^ To further enhance the osteoconductivity and biological properties of ADM scaffolds, researchers have explored incorporating bioactive molecules, such as β-tricalcium phosphate (β-TCP), into the metal matrix.^120^ Doping metal materials with bioactive molecules gives rise to a new class of biomaterials known as “doped metal materials” for ADM. Among the various doped metal materials for ADM, zinc (Zn) has emerged as an up-and-coming candidate due to its unique combination of properties.^121,122^ Zn is an essential mineral for bone health and has been shown to promote osteoblast proliferation and differentiation, the critical processes involved in bone regeneration. Moreover, Zn ions exhibit antibacterial properties that can help to prevent infections associated with implant surgery. Zinc-doped metal scaffolds have demonstrated promising results in preclinical studies, demonstrating their ability to promote bone regeneration and enhance healing. Using this ADM technique, authors could generate scaffolds composed of high amounts of BCP powders (45 vol %) containing different HA/β-TCP ratios in the presence of a cross-linked polymer. Furthermore, the nonexistence of a sintering step allowed for incorporating levofloxacin, an antibiotic, into the scaffolds to treat bacterial infections simultaneously with bone regeneration. Additionally, the use of metallic foams could be a solution to improve mechanical resistance and promote osseointegration of large porous metal devices. In a recent study, titanium cylinders were prepared by ADM (3D printing/rapid prototyping) with a geometric or trabecular microarchitecture (Figure 4B).^115^ They were implanted in the femoral condyles of aged ewes; the animals were left in stabling for 90 and 270 days. Notably, bone anchoring occurred on the margins of the cylinders, and some trabeculae extended into the core of the cylinders, but the amount of bone inside the cylinders remained low. The rigid titanium cylinders preserved bone cells from strains in the core of the cylinders. The authors mentioned that while ADM is an exciting tool for preparing 3D metallic scaffolds, the microarchitecture does not seem as crucial as expected, and anchoring seems limited to the first millimeters of the graft.^123,124^

## 4 Nanostructured Biomaterials for Superior Bone Regeneration

**Figure 5:**
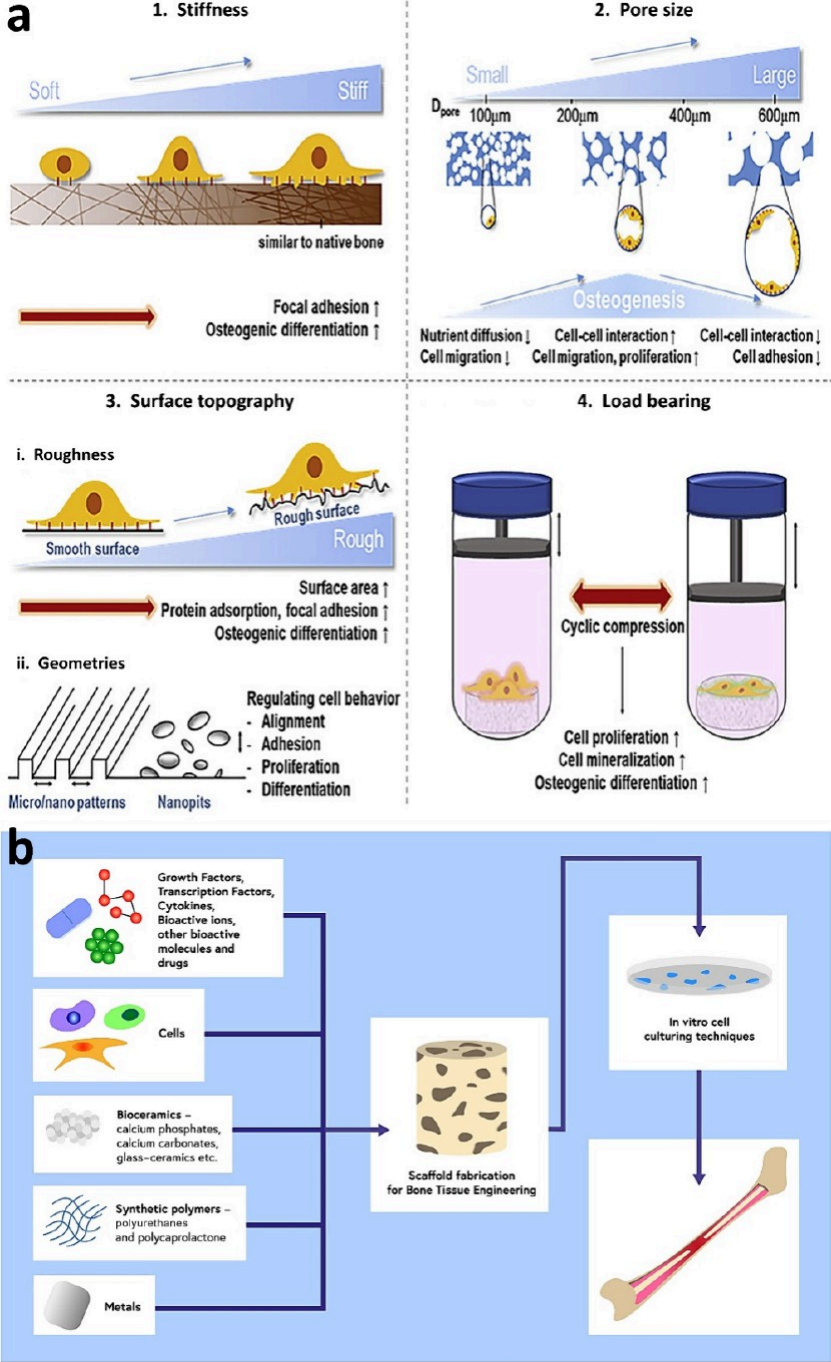
(a) Schematic illustration of scaffold properties for bone-tissue engineering to enhance osteogenic differentiation, and the image provides a visual representation of how these scaffold properties contribute to the process of bone regeneration.^129^ Reproduced with permission. Copyright 2023, Elsevier. (b) Strategies to improve the BTE material’s effectiveness and showing approaches and advancements in BTE materials, offering insights into how these strategies can improve bone regeneration outcomes.^137^ Copyright 2022, MDPI, Basel, Switzerland (Creative Commons (CC BY) license).

Most recent BTE research focuses on composite multiphase materials consisting of two or more components (e.g., hydrogels, nanofiber scaffolds, and 3D printing composite scaffolds) with varying morphology or composition.^125,126^ Such biomaterials offer synergistic properties that are not achieved from each component alone. They are known for enhanced biological characteristics and improved performance for bone regeneration by leveraging the advantages of combining multiple materials, addressing the limitations of individual materials in terms of biological, physical, and chemical properties.^127,128^ Using novel complexes (e.g., scaffolds) instead of simple bone grafts is to “mimic” the structure and function of natural bone and its ECM. The scaffolds provide a three-dimensional setting to encourage cell attachment, growth, and proliferation while possessing suitable physical properties for bone regeneration.^129^ Properties such as porosity,^130^ surface topography,^131^ stiffness,^132^ and load-bearing capacity^133^ are crucial in designing ideal BTE materials (Figure 5A). For example, Woodard et al. showed that a gradient, multiscale porous scaffold with micro- and macropores exhibited enhanced osteoconductivity compared to a scaffold characterized by macropores only. Scaffolds’ micropores could effectively retain more growth factors within the structure.^134^ For instance, Andrukhov et al. modified the roughness of the titanium scaffold using sandblasting and acid-etching methods, which enhanced the proliferation and osteogenic differentiation of the cells seeded on the material’s surface.^135^ To prove the importance of stiffness in designing the BTE material, Chen et al. designed 3d decellularized bone material with varying stiffness levels (from 13 to 37.7 kPa). The materials maintained the identical bone microstructure while coating with different collagen and HAP ratios. The *in vitro* and *in vivo* findings verified that the scaffolds with the highest level of stiffness exhibited the most pronounced osteogenic differentiation, cell recruitment, and angiogenesis.^136^ Moreover, integrating the scaffolds with osteoinductive cues, such as drugs, natural pharmaceutical compounds, growth factors (GF), MSC, microRNAs, and other inorganic ions, has been proven to be effective in enhancing material functionality and effectiveness^137,138^ (Figure 5B). Small molecule drugs and other active compounds, including statins,^139^ antibiotics,^140^ dexamethasone,^141^ adenosine,^142^ aspirin,^143^ etc., have demonstrated advantages in promoting bone regeneration despite not all specifically targeting bone tissue. Natural pharmaceutical compounds also possess significant potential in the regeneration of bone tissues. Investigations have indicated the positive contribution of curcumin,^144^ icariin,^145^ cannabidiol,^146^ etc., to bone regeneration. Among many GF existing in the human body, we can mention bone morphogenic proteins (BMPs),^147^ VEGF,^148^ BGP,^149^ etc., as active proteins or polypeptides that regulate the growth and development of bones. Additionally, it is worth mentioning that using microRNA as an osteoinductive cue is expected to emerge as an alternative strategy in BTE. Many microRNAs (miRNA-26a, −135, or 138b) control factors specific to bone development, osteoblast differentiation, and osteoporosis pathology.^138,150^ Hydrogel scaffolds have also attracted significant interest in the bone regeneration field due to their similarity to ECM (shown in Figure 5B), the composition of a highly interconnected hydrophilic network with porous structure, favorable biocompatibility, and the capacity to stimulate bone restoration.^151^ Hydrogels effectively treat bone defects, promoting osteoblast differentiation and proliferation, enhancing angiogenesis, modulating immune response, and facilitating mineralization.^152^ However, the main hydrogels’ limitations are poor mechanical properties, uncontrollable biodegradation, and low stiffness.^153^ Given the above, additional enhancements are necessary to improve these properties, including incorporating sacrificial bonds, forming more homogeneous networks, creating double-network hydrogels, or incorporating inorganic fillers.^151,154^

### 4.1 Electrospinning of Nanostructured Fibrous Platforms for Bone Regeneration

**Figure 6:**
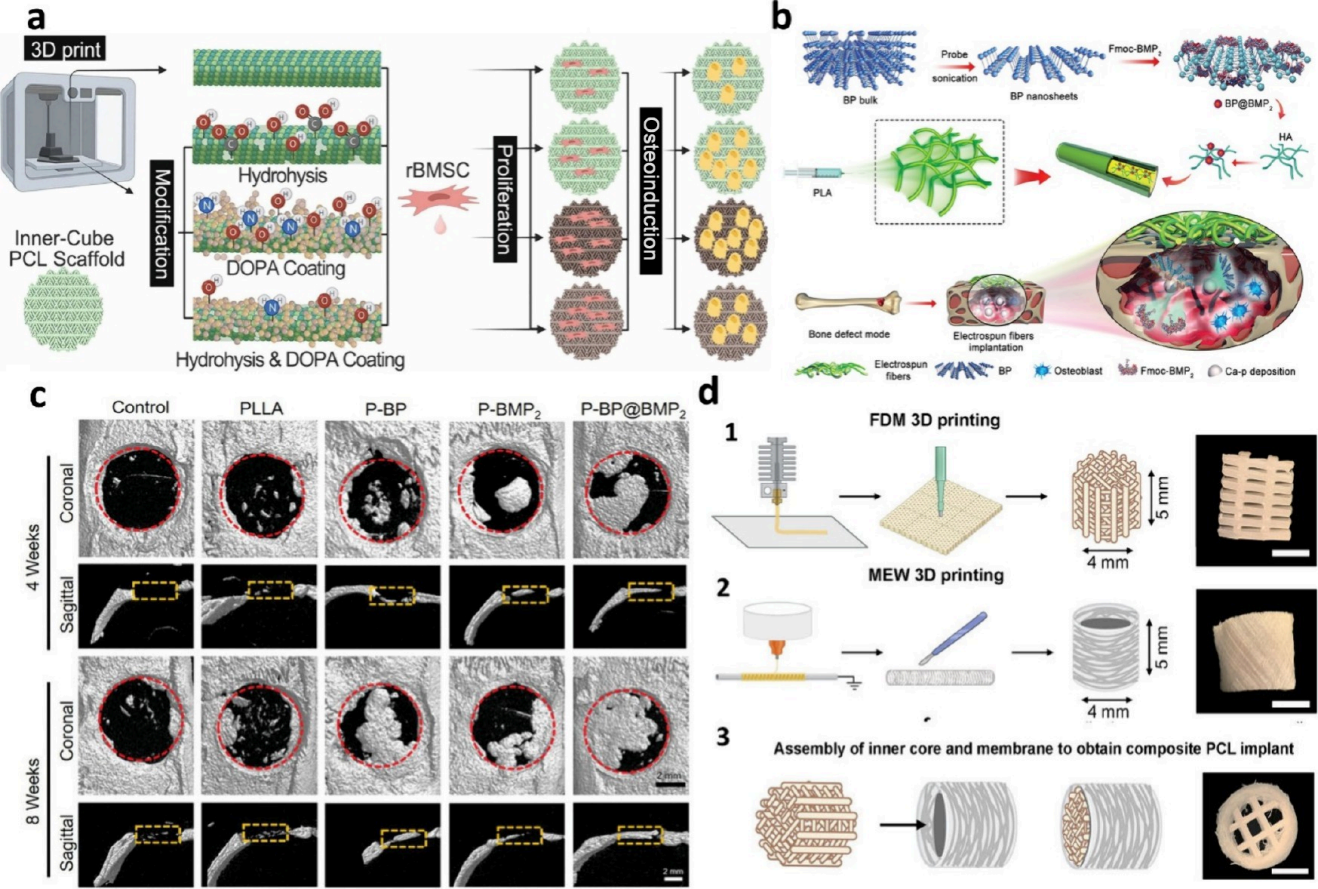
(a) Functionalization of 3D printed polycaprolactone by bionic hydrolysis and PDA coating as well as the combination of these two approaches. All the modifications have significant impact on proliferation and osteogenesis of rat bone marrow mesenchymal stem cells.^166^ (b) A schematic depiction of the process for creating BP@BMP2 electrospun fibrous scaffolds, highlighting their role in attracting osteoblasts and delivering calcium-free phosphate therapy to enhance bone physiological regeneration in vivo.^167^ (c) Radiological analysis in vivo and micro-CT reconstruction images in coronal and sagittal views at 4 and 8 weeks across different groups, with the initial boundary of the critical cranial defect indicated by red dotted lines.^167^ (d) Schematic illustration of the 3D PCL implant. (1) 3D scaffold printed using FDM and bright field image of the final inner scaffold after extraction; scale bar = 2 mm. (2) 3D scaffold printed using MEW and bright field picture of the final mimetic periosteum; scale bar = 2 mm. (3) Schematic of inner implant core and MEW membrane assembly with bright field image of the whole implant; scale bar = 2 mm.

Electrospinning is a cutting-edge technology that has gained considerable recognition in the field of tissue regeneration, particularly in the context of treating bone defects.^155^ This technique involves the controlled deposition of polymer fibers onto a substrate using an electrostatic field. In the realm of bone regeneration, electrospinning offers unique opportunities for designing structures that facilitate the healing and reconstruction of bone tissue. The design of electrospun scaffolds for bone regeneration involves a multidisciplinary approach, integrating materials science, biology, and engineering principles to create platforms that effectively support the regeneration process. Researchers continue to explore new strategies and materials to improve the performance of electrospun scaffolds for various tissue engineering applications.^156,157^ Nanofibers are characterized by a high surface-to-volume ratio suitable for cell attachment and the highest morphological similarity to the ECM,^158^ which is the complex network of proteins and carbohydrates providing structural and biochemical support to cells.^159^ Moreover, electrospun materials possess unique features, such as precise structural design and the ability to incorporate various bioactive substances. A few years ago, it was shown that arranged nanofibers can change the morphology, functions, and direction of cell migration.^160^ However, Zhang et al. reported for the first time the impact of the morphology of carbon nanofibers on bone cells.^161^ Because bone is an electroactive tissue, researchers fabricated electroconductive, polyacrylonitrile (PAN)-based aligned carbon nanofibers (CNFs) as a scaffold for bone regeneration. PAN is interested in this field due to its high carbonization efficiency and appropriate mechanical properties. The studies confirmed the biocompatibility of the scaffolds and indicated that an osteoblast-like cell line (MG-63) grew parallel to the axes of the aligned CNFs, while growth on random CNFs had no specific pattern.^162^ Xia et al. used an electrospinning technique to prepare a membrane that mimics the unique properties of the skull base structure.^163^ The asymmetric layer-by-layer spun membrane contained a superhydrophilic osteogenic polycaprolactone/gelatin-polydopamine (PG–PDA) part. Polydopamine placed in the hydrogel constituting the lower layer significantly supported bone tissue regeneration by inducing hydroxyapatite mineralization *in situ*.^164^ The second layer was a hydrophobic PCL mat used to prevent cerebrospinal fluid leakage and serve as a barrier to avoid the invasion of the surrounding fibrous connective tissue into the bone defects (Figure 6B).^163^ Other scientists have attempted to give PCL/gelatin composite membranes antibacterial properties necessary for the effective operation of guided bone regeneration (GBR), e.g., in the treatment of periodontitis. Wang and co-workers loaded electrospun PG nanofibers with bioactive gold nanoparticles (AuNP) and quantum dots (CD) synthesized using ornidazole as substitutes for growth factors and antibiotics (Figure 6A).^165^ A fibrous membrane provided a scaffold to support the recruitment, proliferation, and differentiation of hPDLSC stem cells, ultimately resulting in coverage of the rats’ bone defect area *in vitro*. The synergistic effect of the PG-AuNP-CD membrane provided the system with excellent osteogenic and antibacterial properties, making it suitable for use as a GBR membrane in a clinical setting.^165^

Another approach using electrospun nanofibers was presented by Vinikoor et al.^168^ Researchers developed a biodegradable injectable piezoelectric hydrogel made of short cryo-cut electrospun poly-L-lactic acid (PLLA) nanofibers embedded in a collagen matrix. Because electrical currents/charges in cartilage are created naturally when joints move or deform, external electrical stimulation may be beneficial in cartilage repair. The prepared material could be implanted into cartilage defects to avoid invasive implantation surgery, and ultrasonic activation allowed for the acceleration of the treatment of osteoarthritis. In addition, such a fabricated system provides a porous aqueous environment that facilitates cell ingrowth and regeneration of damaged tissues. *In vitro* data showed that the developed system could enhance cell migration and induce stem cells to secrete TGF-β1, which promotes chondrogenesis. Studies on rabbits *in vivo* confirmed increased subchondral bone formation, better structure of hyaline cartilage, and mechanical properties similar to healthy native cartilage.^168^

### 4.2 3D Printing and Patient-Specific Implants

3D printing, also known as additive manufacturing (AM), is a process that creates three-dimensional objects from a digital model.^169^ Unlike traditional subtractive manufacturing methods that involve cutting or shaping material to create a product, 3D printing adds material layer by layer to build the final object.^170^ 3D printing technology has made significant advancements in the field of medicine, particularly in the creation of patient-specific implants for bone regeneration.^169^ This innovative approach enables the fabrication of implants that precisely match the patient’s anatomy, ensuring a better fit and alignment, thus offering several advantages over traditional implant methods.^171^ One of them is undoubtedly designing complex geometric structures that mimic the natural architecture of bone. This is particularly beneficial for promoting bone ingrowth and integration with the surrounding tissue. Fabricated implants must be well-tolerated by the body, reducing the risk of rejection or adverse reactions. Therefore, ensuring the optimal combination of biocompatible materials and structural integrity is crucial. Continued research and technological advancements contribute to further improvements in this field. Researchers are exploring integrating bioactive materials and growth factors into 3D-printed implants. These additives can stimulate bone regeneration and enhance the healing process.

Due to its low melting point, PCL is one of the plastics that can be used in various printing methods. The high biocompatibility of this polymer ensures complete absorption into the transplanted tissue, resulting in fully regenerated bone tissue.^172^ Romero-Torrecilla et al. developed a three-dimensional PCL membrane acting as a mimetic periosteum - a carrier of vital regenerative signals for damaged bone.^173^ The implant consisted of an internal and external 3D PCL scaffold, differing in functionality and structure. The internal scaffold was printed using the fused deposition method (FDM) to ensure mechanical stability. The outer scaffold, forming a mimetic periosteal membrane, was printed using the melt electro-writing (MEW) technique. (Figure 6C). Scientists have shown that 3D-printed periosteum is susceptible to functionalization. Mimetic periosteum functionalized with recombinant human growth factor BMP-2 (rhBMP-2) exhibited osteoinductive properties *in vitro* and promoted highly efficient bone regeneration *in vivo*, drastically reducing the effective dose of morphogen. Once rhBMP-2 was functionalized and combined with mesenchymal progenitor cells, the modified periosteum enabled the delivery of both therapies to the injured tissue.^173^ Despite numerous advantages, polycaprolactone has some limitations, such as hydrophobicity and low biological activity. However, surface modification can improve the biological properties of PCL scaffolds, which was confirmed in the work of Lin and co-workers.^166^ In this study, 3D printed PCL scaffolds with controllable microscale stereoporous structures were surface treated using bionic hydrolysis and PDA coating–individually and in combination (Figure 6D). Surface treatment led to increased surface roughness and improved physical and chemical properties of the PCL scaffolds, which in turn increased their biological performance in bone regeneration. However, the PDA coating showed the best properties in promoting rat MSC’s adhesion, proliferation, and osteogenesis *in vivo*.^166^ Titanium (Ti) and its alloys are known for their exceptional corrosion resistance and mechanical strength, so they are widely used in producing clinical implants.^174^ Several studies have shown that changing the surface structural morphology of Ti alloys can improve their bioactivity, particularly by improving bone regeneration and integration.^175^ Wang et al. developed seven Ti_6_A_l4_V implants based on various surface preparation methods and postprocessing technologies, including electron beam melting (EBM) printing and SLM printing–two representative AM techniques.^176^ Scientists investigated the impact of the production process and postprocessing technology on osteogenic activity, bone integration efficiency, and mechanical strength of the implant. *In vitro* studies revealed that 3D-printed implants with regular pore structures were more conducive to the osteogenic differentiation of hBMSCs (human bone marrow mesenchymal stem cells), which was attributed to the skeletal structure of these materials. However, both EBM and SLM printed products contain metal powder residues that, if not properly processed, can cause severe damage to human bone tissue. On the other hand, excessive pursuit of a “powder-free” state in the SLM method will damage the mechanical properties of the implant.^176^

## 5 Biologicals in Bone Regeneration

The bone marrow microenvironment is a dynamic area composed of various cell types, such as osteoblasts, osteoclasts, and immune cells.^177^ It also contains a stroma compartment containing MSC and their differentiated progeny of adipocytes and osteoblasts, as well as endothelial cells, pericytes, and neuronal cells.^177^ A complex network of signaling pathways is involved in MSC development, but only a few MSC signaling pathways have been explored thus far.^178^ Among the explored MSC signaling pathways, several are influenced by growth factors (GFs), hormones, and cytokines.^179,180^ Bone regeneration has been significantly enriched by integrating biological factors into biomaterials, leveraging the body’s natural healing mechanisms. This involves applying growth factors, cytokines, and other bioactive molecules to facilitate and enhance the osteogenic potential of biomaterials.^179−181^ Biomaterials, increasingly prevalent in modern research, excel in transmitting critical biophysical cues essential for tissue engineering.^179,182^ Critical elements in the design of these materials, such as stiffness, pore size, porosity, and topography, along with stress relaxation properties, play a fundamental role in directing tissue formation and regeneration.^179,180,182^ These characteristics are pivotal in modulating cell interactions and extracellular matrix interactions. Moreover, biomaterials offer the advantage of being infused with various biologicals like GFs. GF can be incorporated into the biomaterial to modulate cells’ behavior and improve their survival and outgrowth.^183^ This integration not only enhances the stability of the GFs but also meticulously regulates their release into the extracellular environment, facilitating optimal uptake by cells.^184^ This dual functionality of biomaterials, one providing physical scaffolding and the second controlled biochemical signaling, marks a significant stride in the field of regenerative medicine and tissue engineering.^179^

Principal types of growth factors crucial for bone regeneration are fibroblast growth factors (FGFs), bone morphogenetic proteins (BMPs), VEGFs, insulin-like growth factors (IGFs), and transforming growth factors β (TGFβs).^185^ One of the most abundant GF presents in the bone matrix is the TGFβ family of proteins composed of TGFβ1, TGFβ2, TGFβ3, and all BMPs.^178^ The regulatory role of the TGFβ family in the development of MSCs is widely recognized. For example, TGFβ1 attaches to MSCs during bone remodeling, triggering their migration to the specific site and fostering their differentiation into chondrocytes and osteoblasts.^178,186^ For example, Yang et al. fabricated a novel 3D-printed scaffold incorporating transforming growth factor-β3 (TGF-β3) and decellularized extracellular matrix for cartilage repair. These scaffolds significantly enhanced mesenchymal stem cell recruitment, differentiation, and chondrogenesis, both *in vitro* and in sheep models.^187^ Another abundantly available GF in the BM microenvironment is IGF-1, which has been proven to be involved in the proliferation and osteogenic differentiation of MSCs.^178^ Additionally, this growth factor is primarily in the mineralization of cells by activating the mTOR pathway.^178,188^ This indicates its possible dual role in MSC regulation. Choi et al. developed a titanium-adhesive polymer nanoparticle system for the dual release of osteogenic growth factors BMP-2 and IGF-1, aimed at enhancing bone regeneration.^189^ The system uses a poly(l-lactide-*co*-glycolide)-grafted hyaluronic acid (PLGA-HA) copolymer with catechol groups for solid adhesion to titanium surfaces. These nanoparticles showed excellent loading capacity for BMP-2 and IGF-1, and the dual release of these growth factors significantly enhanced the osteogenic potential of human adipose-derived stem cells.^189^

VEGF plays a crucial role in regulating angiogenesis during bone healing, with its levels notably increasing in the initial phase following a bone fracture.^185^ The increase in VEGF levels at a fracture site responds to lower oxygen levels. This change is detected by the hypoxia-inducible factor, stimulating VEGF production. VEGF promotes the growth and migration of endothelial cells, crucial for new blood vessel formation. It also helps attract and sustain cells vital for bone formation. This increase occurs as a response to the reduced oxygen levels at the fracture site, detected by the hypoxia-inducible factor, which stimulates VEGF production, leading to new blood vessel formation. VEGF actively encourages the growth and movement of endothelial cells, which are essential for creating new blood vessels, and it also aids in attracting and maintaining cells responsible for bone formation.^179,180,190^ In the study, Tang et al. developed a dual-modular scaffold for enhanced bone regeneration, using a two-part system to deliver growth factors.^191^ The first module, made of mesoporous bioactive glass, is functionalized for the slow release of bone morphogenetic protein-2 (BMP-2), fostering osteogenesis. The second module incorporates GelMA hydrogel columns for the targeted delivery of VEGF, stimulating angiogenesis. This innovative design allows for synergistic bone growth and vascularization promotion.^191^ The scaffold’s unique structure and functionalization demonstrate promising potential for applications in bone tissue engineering.^191^ The critical role played by FGFs in the development and regeneration of various tissues is noteworthy, particularly FGFs 2, 9, and 18, which play a significant role in bone regeneration.^192^ The Zhang et al. study investigated the effect of FGF-2-induced human amniotic mesenchymal stem cells (hAMSCs) seeded on a human acellular amniotic membrane (HAAM) scaffold for tendon-to-bone healing. *In vitro* and *in vivo* experiments, including a rabbit model, demonstrated that this combination accelerated healing, showing better results in bone tunnel narrowing, higher macroscopic and histological scores, and enhanced mechanical strength compared to other treatments.^193^ Cytokines, including interleukins like IL-1β, IL-6, and tumor necrosis factors (TNFα), play a crucial role in the inflammatory phase of bone healing.^178^ They help orchestrate the immune response, aiding debris removal and preparing the site for new bone formation.^194^ These cytokines, secreted by macrophages within the first 24 h of bone damage, initiate repair and regeneration processes, such as the induction of angiogenic and growth factors.^195^ TNF-α, in particular, activates osteoclasts for debris removal and helps recruit MSCs.^196^ However, the overexpression of these cytokines can cause chronic inflammation and hinder healing.^197^ For instance, while IL-1β is beneficial initially, prolonged exposure can inhibit the osteogenic differentiation of MSCs.^197^ In chronic inflammation, high IL-1β levels impair MSCs’ ability to become osteoblasts, thus affecting bone regeneration.^197^ Lackington et al. investigated a gene therapy approach for bone healing that mitigates the adverse effects of IL-1β. They used a collagen-hydroxyapatite scaffold to deliver nanoparticles containing plasmid DNA for the IL-1 receptor antagonist (IL-1Ra). This strategy showed potential in protecting bone marrow-derived MSCs from IL-1β’s inhibitory effects on osteogenesis, demonstrating the complex role of IL-1β in bone healing. It is beneficial in early stages but harmful if prolonged, making antagonists like IL-1Ra helpful in therapeutic strategies.^197^ Small bioactive molecules have recently been considered an alternative to growth factors, hoping that these will be better suited for regenerative medicine. Such molecules like nitric oxide, oxygen, etc., are more stable than traditional growth factors.^179^ Research suggests that incorporating these osteogenic small molecules into scaffolds can profoundly modify the behavior of MSCs,^179,180^ significantly boosting bone regeneration efforts. Oxygen stands out as a critical component for cell survival, growth, metabolism, differentiation, and intercellular communication.^180,198^ Normally, cells are well-supplied with oxygen via capillaries. However, distances exceeding 100–200 μm from blood vessels can lead to hypoxia, a condition typically triggered by disruptions in the vascular network at injury sites.^180,199^ This results in delayed oxygen transport and can culminate in widespread cell death and tissue necrosis, particularly in skeletal cells that are highly dependent on oxygen due to their intense metabolic demands.^180,199^ Thus, ensuring adequate oxygen supply to these tissues and adapting cellular metabolism to hypoxic environments is essential.^180,199^ Tissue engineering is at the forefront of developing innovative solutions for tissues to produce oxygen within tissues directly to produce oxygen within tissues. This strategy involves integrating oxygen-generating substances into biomaterials. A significant innovation in this area is the composite hydrogel developed by Sun et al., designed to transform reactive oxygen species (ROS) into oxygen.^198^ This hydrogel dynamically adjusts its oxygen production in response to the specific ROS levels in the affected area, efficiently generating oxygen as needed. This not only ensures a steady oxygen supply but also fosters angiogenesis, highlighting the hydrogel’s potential to enhance tissue regeneration.^198^

A multidisciplinary approach is becoming increasingly crucial for advancements in regenerative medicine, particularly in bone regeneration. This methodology integrates cell biology, materials science, and engineering expertise to create novel biomaterials and treatment approaches. Growth factors, cytokines, and tiny bioactive compounds have been integrated with biomaterials to broaden the scope of possible treatments and improve our comprehension of tissue dynamics and cellular processes. Furthermore, the use of gene therapy and drug delivery systems to promote bone regeneration is growing. With the help of these techniques, the release of medicinal drugs can be precisely controlled, increasing their efficacy and reducing any possible adverse effects. For long-term tissue regeneration, scaffold systems and nanoparticles engineered to release cytokines and growth factors under control can offer long-lasting therapeutic effects.^178,180^

### 5.1 Growth Factors in Bone Repair

For many years, the evolving landscape of bone tissue regeneration has been focused on therapeutic growth factors like BMP-2 in orthopedic and dental procedures, including spinal fusion and bone augmentation;^203^ while these treatments offer alternatives to traditional bone grafts, they are not without complications.^181^ The challenges of using high concentrations of BMP-2 can lead to adverse effects like excessive inflammation and ectopic bone formation.^204^ The recent shift toward controlled release mechanisms, such as hydrogels, to mitigate rapid release and its associated risks represents a significant advancement in regenerative strategies, aiming to replicate the healing efficiency of autografts while minimizing side effects.^205^ However, besides recombinant GF local delivery, GF can be delivered indirectly by injecting Platelet Rich Plasma^206^ or by using cell therapies.^207^ As was shown in previous examples, novel strategies for bone tissue engineering involve multiple approaches, including scaffolding, cells, growth factors, and small molecule delivery. Hydrogels releasing GF sustainably can help increase the availability of bioactive GFs due to their rapid degradation *in vivo*, short half-life in physiological conditions, and deactivation by enzymes. A hydrogel recapitulating a growth factor-enriched microenvironment for bone regeneration was reported by Zhang et al.^208^ The sulfated gelatin (S-gelatin) hydrogel released bone morphogenetic protein-2 (BMP-2) to direct MSC differentiation, stimulate cell proliferation, and improve bone formation. The S-Gelatin amplified BMP-2 signaling *in vitro* in mouse MSCs by enhancing the binding between BMP-2 and BMP-2 type II receptors (BMPR2). The receptor activation affected MSC response and cytokine secretion promotion, enriching endogenous growth factor secretion and enhancing vascularization in mice models. Hydrogels, in general, by encapsulating growth factors within their matrices, can protect them from quick enzymatic breakdown or deactivation and slow their release.^205^ A benefit of using sulfated gelatin was the possibility of electrostatically interacting with positively charged BMP and creating a regenerating microenvironment critical for tissue repair. Chen and colleagues developed a polyhedral oligomeric silsesquioxane (POSS)–modified porous gelatin hydrogel.^209^ This was achieved by reacting the amine groups in POSS with the carboxyl groups of gelatins, aiming to facilitate vascularized bone regeneration in calvarial defects. The hydrogel was used to deliver VEGF and BMP-2, augmenting its therapeutic effectiveness. The inclusion of POSS reduced the hydrogel’s pore size and increased its mechanical strength by providing numerous cross-linking points within the hydrogel matrix. *In vitro* study confirmed that the POSS network improved the attachment and growth of rat bone marrow stromal cells (rBM-MSCs) and human umbilical vein endothelial cells (HUVECs) on the hydrogels, also enhancing the hydrogels’ angiogenic support capabilities. Additionally, hydrogels containing 3 wt % POSS and those without POSS consistently released BMP-2 and VEGF attached to their surfaces over 4 weeks. The 3% POSS hydrogel, when combined with growth factors and seeded with rBM-MSCs, promoted vascularization and bone regeneration in a critical-sized calvarial defect rat model, outperforming its counterparts without growth factor coupling (both 3% and 0% POSS hydrogels). This study highlights the effective role of combining VEGF and BMP-2 in inducing vascularized bone regeneration. It underscores the significance of POSS as a key component that synergistically works with growth factors in hydrogel-based systems to enhance bone healing.

Another innovative approach to bone regeneration was presented by Kim et al. in the form of an injectable poly(organophosphate) hydrogel scaffold (IPS) that encapsulates two key growth factors: bone morphogenetic protein (BMP)-2 and TGFβ-1 (IPS_BT).^210^ This system mimics the natural bone healing process, where growth factors are released precisely, time- and concentration-dependently. The IPS_BT system uniquely allows for the slow release of TGFβ-1 while retaining BMP-2 for an extended period. This dual-growth factor release pattern was achieved without requiring multiple materials or complex scaffold designs. When injected *in vivo*, the sol formed hydrogel at body temperature and gradually replaced bone tissue. The study also highlights that in the early stages of bone regeneration, angiogenic markers (CD31 and alpha-smooth muscle actin (α-SMA)) and stemness markers (Nanog and SOX2) are significantly upregulated. Following the *in vivo* administration of IPS_BT, a sequence of angiogenesis, stem cell attraction, and osteogenesis was observed. In both ectopic and orthotopic settings, IPS_BT effectively enhanced bone regeneration in the area where the hydrogel was injected in a noninvasive way. The presence of stem cells infiltrating and bone tissue forming within the IPS hydrogels *in vivo* indicates that IPS creates a conducive environment for bone regeneration. Rather than uniformly distributing GF in hydrogel systems, alternative carriers can be used to modify GF release or enable the sequential delivery of specific GFs. In this context, Wang et al. utilized PLGA microspheres with a core–shell structure (microcapsules) to encapsulate VEGF-A or BMP-2 using a coaxial channel injection method. To mimic the natural bone healing process, they achieved the staged release of VEGF-A and BMP-2 *in vitro* using PLGA microcapsules with varying molecular weights (Mw) and shell thicknesses. Microcapsules containing VEGF-A with a lower molecular weight were used to induce the formation of lumen structures by vascular endothelial cells at an early stage *in vitro*. Conversely, microcapsules containing BMP-2 were designed to promote osteogenic differentiation, with a delayed effect observed when using PLGA of 150 kDa. The core–shell PLGA microcapsules in their study could release VEGF-A and BMP-2 sequentially at different stages, effectively replicating the natural process of bone repair. Over the years, numerous approaches have been devised to integrate growth factors into biomaterials, compensating for their inherent lack of osteoconductive characteristics.^211^ Techniques that offer a regulated release pattern, particularly those enabling the sequential release of multiple growth factors, are attracting significant attention from researchers. The ability to control tissue growth by managing the localized presence of various growth factor combinations presents a potent method for investigating and influencing a broad spectrum of developmental and regenerative activities, which are crucial in biological and medical fields.

### 5.2 Biomaterials for Gene Delivery

Gene therapy holds substantial potential in regenerative medicine and stands as a promising approach for steering stem cell differentiation. Despite successfully utilizing both plasmid DNA and RNA, particularly in bone tissue engineering, plasmid DNA encounters hurdles concerning its safety and efficacy. RNA has emerged as an alternative due to its superior transfection efficiency. Notably, in terms of delivery, plasmid DNA requires nucleus entry for transcription, potentially integrating into host DNA and inducing unwanted genetic alterations. In contrast, RNA only necessitates cytoplasmic delivery for transcription and can regulate gene expression to control disease progression.^212^ Considering its application scope, plasmid DNA triggers encoded protein production solely in dividing cells, while RNA can induce this in both dividing and nondividing cells.^213,214^ RNA therapeutics face degradation by nucleases within tissues and cells and experience electrostatic repulsion due to their negative charge, hindering their interaction with negatively charged cell membranes. This leads to inadequate endocytosis and drug escape from endosomes. Thus, the primary challenge in RNA therapy lies in precisely and effectively delivering these molecules to specific tissues and cells. Transfection methods must consider various factors, including the cell membrane’s negative charge, precise cell targeting, molecule stability during cytoplasmic travel, molecule transportation into the cell nucleus, and the amplified expression of the intended gene. To tackle these hurdles, it is imperative to engineer appropriate vectors that efficiently transport RNA therapeutic drugs to target cells. These vectors must facilitate effective escape from endosomes, enabling robust drug expression for optimal therapeutic outcomes.^212^ Viral and nonviral vectors, such as liposomes, cationic lipids, polymers, and proteins, serve as carriers employed for cell transfection.^215−217^ When comparing these two groups, viral vectors demonstrate superior transgene expression and transduction efficiency. Nevertheless, they carry inherent immunological risks, have limited tropism, and face size restrictions for the inserted transgene.^218^

### 5.3 Functionalizing Scaffolds for RNA-Based Approaches

**Figure 7:**
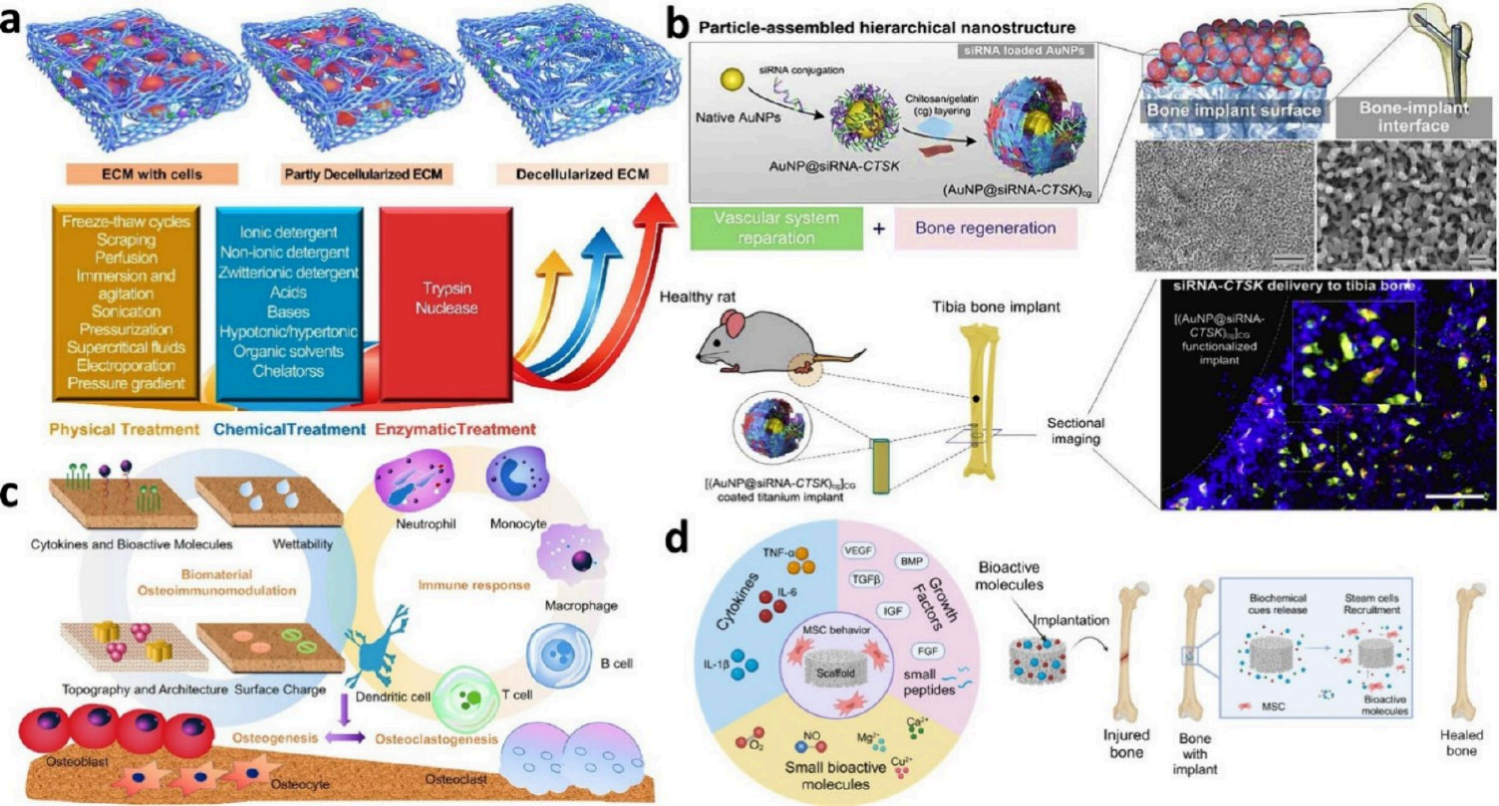
(a) Schematic representation depicts the physical, chemical, and enzymatic treatment stages of tissue decellularization.^200^ (b) Particle-based hierarchical nanostructured implant coatings. AuNPs loaded with siRNA-CTSK were synthesized through a layer-by-layer assembly of biocompatible and antibacterial chitosan and gelatin multilayers.^201^ (c) Approaches focused on changing the topography, wettability, surface charge, and the controlled release of cytokines and bioactive molecules from bone biomaterials.^202^ MiR-29a promotes osteoblast proliferation. (d) Schematic representation of bone regeneration: scaffold implementation with bioactive molecules.

A wide array of RNA therapeutics aimed at bone regeneration have emerged, drawing from the diverse spectrum of RNA molecules such as mRNA (mRNA), miRNA, small interfering RNA (siRNA), and long noncoding RNA (lncRNA).^219^ The dominant class of RNA carriers comprises mRNA molecules, which are naturally synthesized as pre-mRNA in the nucleus, undergo processing, and are then exported to the cytoplasm for translation into proteins by the ribosome’s machinery. Introducing particular mRNA molecules into the cellular cytoplasm allows for synthesizing specific proteins, potentially bolstering bone osteogenesis. For example, adding chemically modified RNA encoding the BMP2 gene demonstrates improved bone regeneration.^220^ Wang et al. demonstrated that MicroRNA-29a signaling shielded against the disruption caused by glucocorticoids on Wnt and Dkk-1 actions, consequently enhancing osteoblast differentiation and mineral acquisition. Improving miR-29a signaling is a viable alternative to alleviate the bone deterioration induced by glucocorticoids.^221^ Research conducted by Zhang et al. revealed that miR-29a facilitated the proliferation of hFOB1.19 cells, contrasting with the inhibitory effect of DKK-1 on their expansion. Furthermore, miR-29a demonstrated the ability to hinder apoptosis in hFOB1.19 cells, whereas DKK-1 exhibited the propensity to induce apoptosis in these cells (as depicted in Figure 7E,F).^222^ Li et al. highlighted the significance of miR-21 in osteoblast differentiation, emphasizing Smad7 as a pivotal regulator of osteogenic differentiation. Smad7 involves inhibiting proliferation, differentiation, and mineralization in mouse osteoblast cells.^223^ In a recent study spearheaded by Xing et al., a specifically designed siRNA targeting cathepsin K was subsequently attached to nanoparticles. These functionalized nanoparticles were assembled onto a bone implant, creating a hierarchical nanostructured coating (Figure 7G). This coating significantly enhances cell viability and the release of growth factors associated with vascularization by regulating mRNA transcription. Additionally, experiments using microchip-based methods demonstrate that the nanostructured coating promotes synergy in macrophage-induced upregulation of at least seven bone and vascular growth factors. Assessments in ovariectomized rat and comprehensive beagle dog models highlight that siRNA-integrated nanostructured coating exhibits all the crucial characteristics of an up-and-coming clinical candidate to address the diverse challenges associated with bone regeneration.^201^

### 5.4 Biologic Insights into Immune Modulation via ECM Decellularization

The evolution in orthopedic biomaterial design has moved from favoring “immune-friendly” materials to “immunomodulatory”. Immunomodulation allows biomaterials to regulate the body’s inflammatory response by steering macrophage polarization within the local immune environment. This precise adjustment fosters the development of bone tissue and streamlines the seamless integration between the implant and the bone, thereby enhancing optimal healing and integration.^224^ When designing bone scaffolds, it is crucial to prioritize direct bone growth stimulation and the implant’s ability to modulate the immune system.^225^ Manipulating immune responses by directing immune cell behavior during the initial stages is a considered approach in advancing bone biomaterials. Among immune cells, macrophages are notable for their rapid recruitment and prolonged presence at regenerative sites. Beyond their phagocytic role, macrophages exhibit high adaptability, capable of polarizing into M1 (pro-inflammatory) and M2 (anti-inflammatory) phenotypes in response to the local microenvironment. Achieving an optimal microenvironment involves regulating M1 or M2 phenotypes through various means, including biomaterial surface features,^226^ chemical compositions,^227^ bioactive molecule incorporation like cytokines,^228^ anti-inflammatory drugs,^229^ artificial ECM, and nucleic acids.^202^Figures 7a and d summarize some osteoimmunomodulatory strategies of bone biomaterials. Lin et al. explore the osteoimmunomodulatory effect of an extracellular bioactive cation (Mg^2+^) in the bone tissue microenvironment through their study involving custom-designed PLGA/MgO-alendronate microspheres. Their findings indicate that the Mg^2+^-regulated tissue environment effectively triggers macrophage polarization, transitioning from the M0 to M2 phenotype. This shift is achieved by boosting the production of anti-inflammatory (IL-10) and pro-osteogenic (BMP-2 and TGF-β1) cytokines. Additionally, this regulated environment fosters a favorable osteoimmune setting, supporting the proliferation and osteogenic differentiation of bone marrow mesenchymal stem cells.^224^ Another interesting example of osteoimmunomodulatory effect was investigated by Garg et al., who delved into the influence of fiber and pore size within an electrospun scaffold on the polarization of mouse bone marrow-derived macrophages (MBMMs) toward either regenerative (M2) or inflammatory (M1) phenotypes. Their study revealed a direct relationship between escalating fiber/pore size and heightened expression of the M2 marker, Arginase 1 (Arg1), coupled with diminished expression of the M1 marker, inducible nitric oxide synthase (iNOS), among BMMFs cultured on these scaffolds. Moreover, cultures utilizing larger fiber/pore size scaffolds exhibited increased secretion of angiogenic cytokines such as VEGF, TGF-β1, and bFGF.^226^ An increasingly prominent trend in bone tissue engineering involves the utilization of decellularized extracellular matrix (dECM) derived from tissues. Figure 7C illustrates a comprehensive schematic diagram detailing the physical, chemical, and enzymatic processes involved in tissue decellularization. Decellularization removes cellular and immunogenic substances while safeguarding the ECM’s natural elements and mechanical properties, which are crucial for oxygen and nutrient transport to organs. Within bone tissue engineering, the widespread utilization of dECM scaffolds is attributed to their three-dimensional configuration, mechanical solid attributes, and osteoinductive qualities akin to those found in natural bone. Bone Extracellular Matrix (bECM) stands as a vital noncellular component within bone tissue, comprising type I collagen, glycoproteins, proteoglycans, and noncollagenous proteins such as osteocalcin (OCN), osteopontin (OPN), osteonectin (ON), and sialoprotein.^230^ Striking the right balance between maintaining ECM structure and removing cellular components is essential for producing optimal dECM scaffolds.^231^ The adaptability of dECM allows its use across various applications, including scaffold forms like powder and a digested solution serving as bioink for 3D printing. In recent advancements, the human bone marrow-derived mesenchymal stem cell-derived microsome (BMTS) shows immense promise as a scaffold for bone tissue engineering. Lee et al. devised this hybrid model, combining an ECM-enriched hydrogel within a PCL scaffold. This model demonstrated exceptional viability of BM-MSCs and notable osteogenic activity *in vitro*.^232^ Guler et al. recently conducted a study with the primary goal of pioneering the functionalization of a PGS elastomer using a decellularized bone matrix. This approach aimed to generate an osteoinductive scaffold that effectively promotes robust osteogenesis in bone marrow-derived MSCs.^233^

## 6 Transition from Lab to Clinic: Addressing Regulatory Challenges

For successful clinical translation, biomimetic scaffolds for bone tissue engineering must meet several criteria, including FDA approval, cost-effective manufacturing, sterilization feasibility, easy handling, radiographic distinguishability, and minimally invasive implantation.^234^ The roadmap for translating biomaterials from concept to product finds its initial point in academic research and ends up with product commercialization.

**Figure 8:**
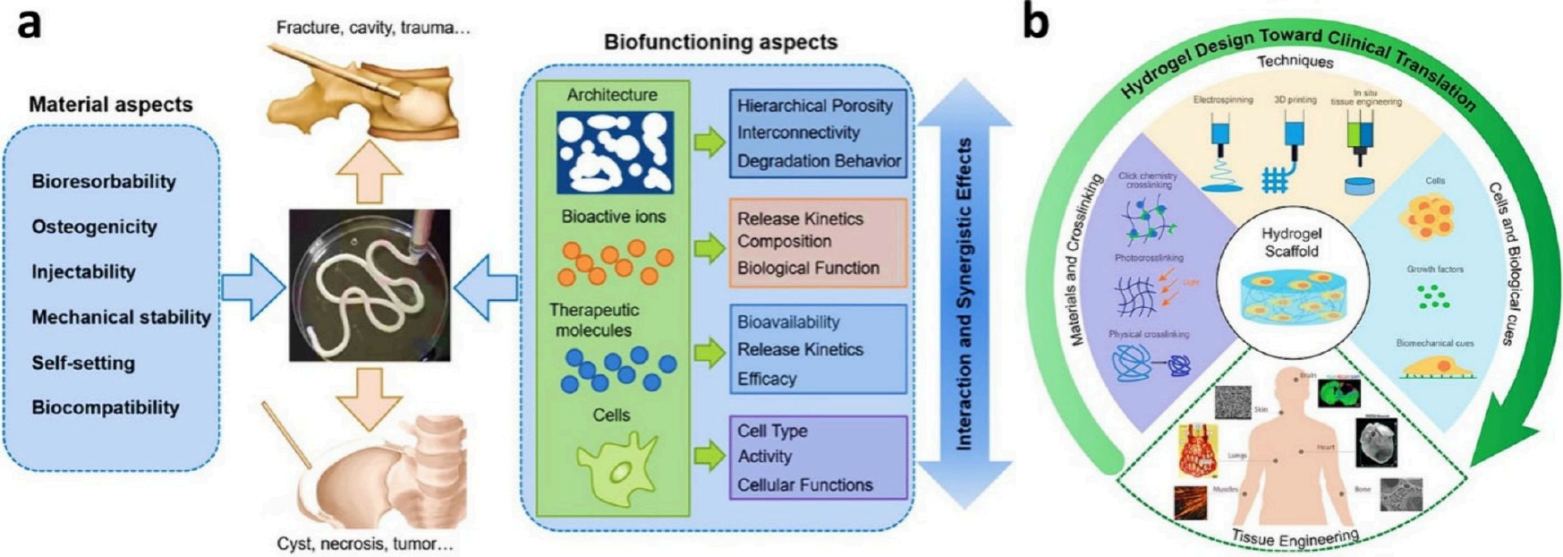
(a) Properties and characteristics of bioceramic cement for effective translational research.^241^ (b) Schematic overview of the necessary steps for HA and CS hydrogels to achieve clinical translation and design of functional hydrogels for tissue engineering. Selected constituent images reproduced with permission from references.^244,243^

Key properties of bone scaffolds, including biocompatibility, biodegradability, osteoinductivity, pore structure, grain size, and surface topography, are crucial for successful clinical treatments. The scaffold must not trigger an immune response, degrade appropriately, and recruit osteoprogenitor cells for bone regeneration.^235^ Thus, establishing and improving the fabrication process is essential for ensuring biomaterials research’s authenticity, reliability, transparency, and reproducibility.^236^ However, some barriers hinder the translation of bone scaffolds to clinical applications. These barriers involve preclinical, clinical, commercial, and regulatory domains (Figure 8A).^237^ In this frame, two of the most significant challenges are the lack of accurate preclinical models and the complexities of clinical trial design. These include challenges related to timing of assessment, short-term and long-term safety evaluation, surgical procedures, choice of control groups, and effective communication of risks and benefits.^238^ The communication gap between academia and industry, intellectual property considerations, and regulatory challenges further complicate the translation process. Similarly, scalability issues in transitioning the material production from laboratory to large-scale crucially affect the clinical translation pathway. More specifically, while numerous studies in rodents have proven the concept of bone tissue engineering, scaling up to larger animal models poses new challenges, mainly related to oxygen and nutrient availability postimplantation.^239^ Even though rodents offer cost-effectiveness and ease of handling, large animals could provide a more relevant comparison to human conditions. Herein, the selection of an animal model generally depends on factors like functionality, mechanical testing, histology, and biochemical and molecular assays. However, it is worth mentioning that one strategy to expedite the translation of preclinical findings to clinical applications is evaluating the biomaterials through *in silico* models. Lastly, financial aspects—such as the increased cost and risk of product development—also slow the progress. Thus, a proper funding reallocation should be considered. Over 90% of funding for tissue engineering and regenerative medicine goes to fundamental research rather than clinical translation. Thus, efforts should be underway to shift this imbalance and accelerate the translation of scientific research into clinical applications.^237^

Preclinical studies focused on stem cell-based therapies using various bone scaffolds, such as bioceramics and biodegradable polymers, have demonstrated successful bone regeneration in animal models, paving the way for advancing bone tissue engineering to clinical trials.^240^ Bone cements are injectable materials that go from a liquid or viscous state to a solidified state with enhanced mechanical strength. These materials should be bioactive, bioresorbable, and have suitable mechanical properties for hard-tissue repair for orthopedics, oral defect treatments, and plastic surgery. The clinical standard of bone cement is the FDA-approved PMMA, an acrylic cement widely used in millions of surgeries worldwide.^241^ Other bone cement used for bone tissue engineering are ceramic-based inorganic materials, particularly calcium phosphate (CaP), silica, hydroxyapatite ceramics (Figure 8B), and bioactive glasses.^181,242^ These have the advantage of mimicking the bone’s mineral phase, exhibiting good biocompatibility and osteoconductivity. On the other hand, injectable CaP cements are FDA-approved and widely used for bone defect treatments. Bioactive glasses, with varied formulations, interact with bone and soft tissues, inducing hydroxyapatite carbonate formation. Naturally derived polymers have also shown their clinical translation feasibility. For example, Gelatin-Methacryloyl (GelMA), modified through methacrylation, exhibits favorable properties like biocompatibility, enzymatic degradability, and cell adhesion. Furthermore, it meets GLP/GMP requirements. However, challenges exist in ensuring reproducibility, batch-to-batch consistency, and scalability in GelMA production for clinical applications.^243^

## 7 Conclusions and Future Perspectives

In recent years, there have been significant advancements in bone tissue regeneration. However, there are still challenges and limitations that need to be addressed.^245^ Understanding and tackling these obstacles is essential to progress in regenerative medicine and developing effective treatments for bone injuries. By examining these problems and thinking about new solutions, we hope to contribute to the ongoing conversation about the future of bone tissue regeneration. A critical challenge in bone tissue regeneration is the establishment of sufficient Vascularization to support the growth and vitality of new bone tissue.^246^ Inadequate blood supply hampers nutrient delivery and oxygenation, hindering the formation of functional and durable bone structures. The complex interplay between the immune system and regenerating bone tissue poses a significant hurdle. Inflammatory responses can impede natural healing, leading to delayed or suboptimal regeneration.^247,248^ Balancing immune modulation without compromising the body’s defense mechanisms is a delicate yet crucial task. The inherent Variability among individuals, including age, health status, and genetic makeup, complicates the development of universally effective regenerative therapies.^34,249^ Tailoring approaches to accommodate this diversity is essential for achieving optimal outcomes in diverse patient populations. Gene therapy has become a groundbreaking approach in regenerative medicine, allowing us to influence how cells behave on a molecular level. In bone tissue regeneration, gene therapy offers exciting possibilities for controlling critical signaling pathways related to bone formation, blood vessel growth (angiogenesis), and the body’s immune response. By focusing on genes like BMP-2 (Bone Morphogenetic Protein-2) and Runx2, we can precisely regulate the transformation of mesenchymal stem cells into osteoblasts, the cells responsible for building bone tissue. This targeted approach enhances bone formation. Boosting Vascularization by activating angiogenic factors like VEGF facilitates the growth of a strong network of blood vessels, which helps overcome a significant challenge in bone regeneration—ensuring an adequate blood supply to the healing bone tissue. Managing immune response genes allows us to balance creating a pro-regenerative environment and controlling excessive inflammation. This fine-tuning optimizes the healing process during bone tissue regeneration. Challenges such as limited Vascularization, immune response regulation, and achieving ideal biomaterial integration underscore the complexities of bone regeneration. Nevertheless, promise is on the horizon as we tackle these hurdles head-on. Discussing potential future directions holds the potential to revolutionize the landscape of bone regeneration, offering hope for more effective treatments and advancements in regenerative medicine.

This paper thoroughly explores biomaterials employed for bone tissue engineering, shedding light on their diverse applications and intrinsic properties. We have delved into various categories of biomaterials utilized in scaffold construction, including metal matrix composites, polymer matrix composites, ceramic matrix composites, and functional composites, providing a comprehensive overview reflects on the remarkable progress made in biomaterials research over the years, noting how advancements have transformed the once-daunting task of finding materials compatible with living tissue into a feasible reality. Despite these achievements, the ongoing pursuit of refining ideal biomaterials to ensure seamless integration with host tissues highlights the ever-evolving nature of biomaterial science. Additionally, we have discussed the integration of additives such as signaling molecules, stem cells, and functional materials, showcasing their potential to enhance scaffold efficacy significantly. While finding a material compatible with living tissue seemed intimidating decades ago, today’s advancements underscore biomaterials’ pivotal role in bone repair. Nonetheless, ongoing research endeavors seek to refine ideal biomaterials further to ensure seamless integration with host tissues. The promising future for bone regeneration is fueled by the continuous advancement of novel biomaterials and the adoption of innovative strategies. Specifically, it emphasizes the potential of integrating nanotechnology, stem cell science, and interdisciplinary approaches to further propel the field of bone tissue engineering toward discovery and clinical application frontiers. Overall, the conclusion encapsulates the current state of the art in bone regeneration research while offering insights into the exciting prospects.
